# Utility of urinary podocalyxin levels in patients with and without type 2 diabetic nephropathy and its correlation with renal function

**DOI:** 10.1186/s13104-026-07724-7

**Published:** 2026-02-16

**Authors:** Jothivanan Perumal, Vadivelan Mehalingam, Ramesh Ramasamy

**Affiliations:** 1 Department of Medicine, Jawaharlal Institute of Postgraduate Medical Education and Research (JIPMER), 3rd floor, Institute Block, Puducherry, 605006 India; 2 Department of Biochemistry, Jawaharlal Institute of Postgraduate Medical Education and Research (JIPMER), Puducherry, 605006 India

**Keywords:** Urinary podocalyxin, Diabetic nephropathy, Albuminuria

## Abstract

**Purpose:**

Podocalyxin is a urinary protein that may serve as a potential indicator for the early detection of diabetic nephropathy. The objectives of this study were to assess urinary podocalyxin levels in patients with and without type 2 diabetic nephropathy and to determine its correlation with renal function.

**Materials and methods:**

This cross-sectional analytical study was conducted at a tertiary care teaching hospital in south India for 18 months. Study subjects were divided into four groups: non-diabetics, diabetics without albuminuria, diabetics with microalbuminuria, and diabetics with macroalbuminuria. Urinary albumin was detected by the dipstick method in a spot urine sample for all study subjects. Urinary albumin was quantified by nephelometry. The enzyme-linked immunosorbent assay (ELISA) technique estimated urinary podocalyxin levels in all groups.

**Results:**

Urinary podocalyxin levels were elevated in diabetic subjects compared to the control group. There was a positive correlation between urinary podocalyxin and urine albumin-creatinine ratio (UACR) in diabetic study subjects. No correlation was found between urinary podocalyxin and estimated glomerular filtration rate (eGFR). Urinary podocalyxin showed a sensitivity of 85% and specificity of 64% (at a cut-off value of 275.56 ng/mL) in estimating albuminuria in diabetic subjects. Urinary podocalyxin showed a sensitivity of 89% and specificity of 78% (at a cut-off value of 273.38 ng/mL) in estimating eGFR in diabetic subjects.

**Conclusion:**

Urinary podocalyxin levels were elevated in patients with type 2 diabetic nephropathy. It was positively correlated with the urine albumin-creatinine ratio in diabetic subjects. However, there was no correlation with the estimated glomerular filtration rate.

## Introduction

Diabetes is the leading cause of renal impairment globally, leading to dialysis or kidney transplantation [1]. Diabetic nephropathy is a term used to describe specific pathologic structural and functional abnormalities in the kidneys of patients with diabetes mellitus [2]. Approximately 40% of patients with type 2 diabetes mellitus suffer from this microvascular complication of diabetes [3]. Diabetic nephropathy leads to albuminuria and loss of renal function.

Diabetic nephropathy becomes more likely in patients as the years with diabetes progress. Diabetic nephropathy is a result of microangiopathy that leads to proteinuria and loss of renal function.

Albuminuria is a characteristic laboratory finding in patients with diabetic nephropathy [4]. Following albuminuria, there is a decline in renal function. In patients with type 2 diabetes mellitus and essential hypertension, albuminuria is a predictor of poor renal outcomes. Poor glycemic control is an independent predictor of progression to albuminuria and/or end-stage renal disease (ESRD) in normoalbuminuric patients with diabetes. Sustained and prolonged hyperglycemia leads to the formation of advanced glycation end-products (AGEs), oxidative injury and the production of inflammatory and fibrotic factors which leads to renal fibrosis.

However, in the United Kingdom Prospective Diabetes Study (UKPDS), it was found that 60% of study participants who developed functional impairment of the kidneys did not have prior albuminuria. Also, 40% of subjects never developed albuminuria during the study [5].

Hence, finding new biomarkers for diabetic nephropathy may help in early diagnosis and treatment of the condition. In the early stage of diabetic nephropathy, there is a progressive decline in the number of podocytes along with the disappearance of their foot processes and damage to the filtration slit-diaphragm that leads to proteinuria [6]. Consequently, specific proteins secreted by podocytes in urine may serve as potential markers for the early diagnosis of diabetic nephropathy.

Podocalyxin is a CD34-related sialo-mucin protein expressed by podocytes, mesothelium, vascular endothelial cells, hematopoietic stem cells, and platelets [7]. It is a transmembrane protein localised to the apical compartment of glomerular podocytes [8]. It forms a meshwork that supports the capillaries of the glomerulus.

Podocalyxin is essential in the normal development of glomeruli. It is seen to express laterally between cells and in the intercellular spaces between podocytes. It also plays a role in the morphogenesis of podocytes and maintenance of the structural integrity of podocyte foot processes [9].

A study by Zeng et al., showed that intrarenal podocalyxin is a marker of rapid progression of nephropathy, suggesting that this protein is induced in diabetic kidney disease due to podocyte stimulation or overactivation^10^. Thus, urinary podocalyxin may serve as an early marker for diabetic nephropathy.

This study aimed to assess urinary podocalyxin levels in patients with and without type 2 diabetic nephropathy and to find out the correlation of urinary podocalyxin with renal function [defined by estimated glomerular filtration rate (eGFR) and urine albumin-creatinine ratio (UACR)] in patients with type 2 diabetic nephropathy.

## Materials and methods

This cross-sectional analytical study recruited patients attending the Medicine OPD of JIPMER Hospital, Puducherry, from June 2021 to December 2022. The study was approved by the Institute Ethics Committee (Human Studies), JIPMER, Puducherry (Approval number-JIP/IEC/2021/091). All study subjects received adequate information about the study and gave written informed consent for participation in the study.

Patients above 18 years with type 2 diabetes mellitus as defined by the ADA criteria were recruited as study subjects. Healthy individuals with a random blood sugar below 200 mg/dL and no symptoms of diabetes mellitus were recruited as control subjects. Performing a glycosylated hemoglobin (HbA1c) test on control subjects was not possible due to financial constraints.

Patients with urinary tract infections, autoimmune diseases, hypertension, obstructive nephropathy, systemic disorders, and malignancy were excluded from the study. Patients on angiotensin-converting enzyme (ACE) inhibitors, angiotensin receptor blockers (ARBs), and non-dihydropyridine calcium channel blockers (CCBs) were also excluded. Pregnant women and those with gestational diabetes mellitus were also excluded from the study.

Study subjects were divided into four groups-

1. Diabetic patients with normoalbuminuria (urine albumin-creatinine ratio-0-30 mg/g)
2. Diabetic patients with microalbuminuria (urine albumin-creatinine ratio-30-300 mg/g)
3. Diabetic patients with macroalbuminuria (urine albumin-creatinine ratio > 300 mg/g)
4. Controls-Non-diabetic patients without any renal disease

As there are no previous Indian studies on urinary podocalyxin in patients with type 2 diabetes mellitus, this was conducted as a pilot study. Hence, the study subjects were taken as 30 in each group, and the total sample size was 120. Convenience sampling technique was used in this study. The sample size was considered to be 30 in each group due to financial constraints for the processing of the urinary samples. Convenience sampling was used to select subjects since they were recruited from the hospital’s Medicine OPD.

After obtaining approval from the Institute Ethics Committee (IEC), the study was conducted over 18 months.

Type 2 diabetic patients attending Medicine OPD who met the inclusion and exclusion criteria were enrolled in the study. All the study subjects provided informed, written consent for participation. Patients self-reported their duration of diabetes. Weight, height, and blood pressure (BP) were measured for all study subjects. Serum creatinine was estimated if a value was unavailable in the last three months. The estimated glomerular filtration rate (eGFR) was calculated using the 2021 CKD-EPI equation.

A spot urine sample was collected from all study subjects and tested for albuminuria using the dipstick technique. Based on urine albumin levels (by dipstick method), diabetic subjects were divided into the macroalbuminuria group if the dipstick showed > 3 + protein or micro/normo albuminuria groups if the dipstick showed < 3+.

A 2 mL urine sample was collected from all study subjects, centrifuged, and stored in a deep freezer at -20 °C. The stored urine samples underwent estimation for albumin levels. Urinary albumin was estimated using a nephelometry technique using kits provided by Siemens. Urinary creatinine was also assessed using the same method.

Based on the urine albumin-creatinine ratio (UACR), diabetic subjects were divided into two groups-normoalbuminuria (if UACR < 30 mg/g) or microalbuminuria (if UACR 30–300 mg/g).

After urinary albumin estimation, there were four groups-controls (non-diabetic subjects): diabetics with normoalbuminuria, diabetics with microalbuminuria, and diabetics with macroalbuminuria. Urinary albumin levels were estimated by nephelometry, while urinary podocalyxin levels were estimated using the ELISA technique. Abbkine Inc provided ELISA kits.

Continuous variables were expressed as mean with standard deviation or median with quartiles (Q1, Q3), and categorical variables were expressed as frequency with proportion based on the normality of data. The normality of data was tested using the Kolmogorov-Smirnov test. Urinary podocalyxin levels between the groups were analysed using ANOVA or Kruskal-Wallis tests based on the normality of the data. Spearman’s correlation test analysed the correlation between urinary podocalyxin levels with UACR and eGFR.

All statistical analysis was carried out at a 5% significance level, and a p-value of less than 0.05 was considered statistically significant.

## Results

The baseline characteristics of the study subjects are given in Table 1.

**Table 1:** Baseline characteristics of study subjects

Parameter	Controls (*n* = 30)	Normoalbuminuria (*n* = 30)	Microalbuminuria (*n* = 30)	Macroalbuminuria (*n* = 30)	*P* value
Age (years) (Median with quartile Q1, Q3)	35.5 (30, 49.25)	54.5 (46.5, 60)	56 (49.5, 61.25)	51 (45.75, 58)	-
Gender (male) (Frequency %)	14 (47%)	21 (70%)	19 (63%)	20 (67%)	-
BMI (kg/m^2^) (Mean ± SD)	25.2 ± 3.4	24.7 ± 3.3	24.0 ± 3.1	24.0 ± 3.1	0.13
Duration of diabetes (years) (Mean ± SD)	0	5.1 ± 3.4	6.2 ± 4.7	7.1 ± 5.5	< 0.001
Serum creatinine (mg/dL) (Median with quartile Q1, Q3)	0.7 (0.6, 0.9)	0.7 (0.6, 0.8)	0.7 (0.6, 1.0)	0.9 (0.7, 1.1)	0.06
eGFR (mL/min/1.73 m^2^) (Median with quartile Q1, Q3)	107.5 (94.5, 124.7)	107 (97.2, 113.25)	105 (73.5, 112.7)	99.5 (72, 105)	0.008
Urine albumin (g/L)	10.7 (10.7, 20.8)	10.7 (10.7, 14.4)	26.6 (10.7, 59.1)	340 (136, 1025.7)	< 0.001

Among the study subjects, 74 (61.6%) were male and 46 (38.4%) were female. A more significant number of female subjects (16 out of 30) were in the control group.

The eGFR was calculated using the CKD-EPI 2021 formula. The urine albumin-creatinine ratio (UACR) in different groups is shown in Table 2.

**Table 2:** Urine albumin-creatinine ratio (UACR)

Group	Urine albumin-creatinine ratio (UACR)	Quartile (Q1, Q3)
Control	18.9	11.6, 33.6
Diabetic with normoalbuminuria	16.5	13.3, 24.2
Diabetic with microalbuminuria	65.1	43.6, 161.4
Diabetic with macroalbuminuria	666.5	362.9, 1199.3

Urinary podocalyxin values are shown in Table 3.

**Table 3:** Urinary podocalyxin in different groups

Group	Median urinary podocalyxin (ng/mL)	Quartile (Q1, Q3)
Control	275.8	256.0, 292.7
Diabetic with normoalbuminuria	292.6	266.3, 331.8
Diabetic with microalbuminuria	330.1	285.5, 367.5
Diabetic with macroalbuminuria	304.7	290.8, 344.7

**Fig. 1:**
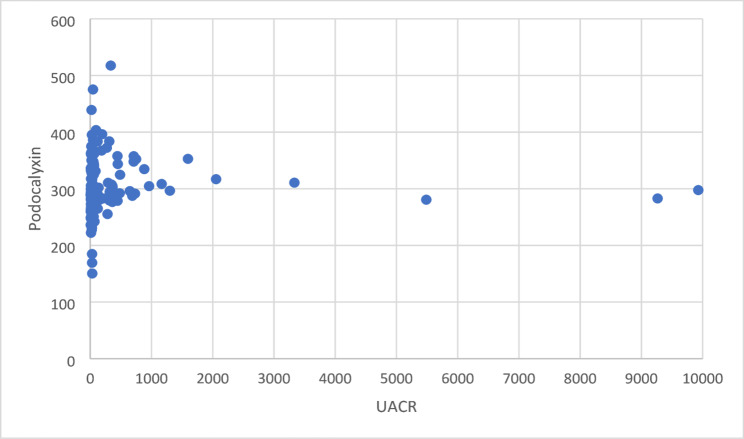
Scatter plot between urinary podocalyxin and UACR

A scatter plot was drawn to study the correlation between urinary podocalyxin and UACR. The parameters were positively correlated (*r* = 0.0265, *p* < 0.01), indicating that urinary podocalyxin increases with an increase in UACR. This is shown in Fig. 1.

**Fig. 2:**
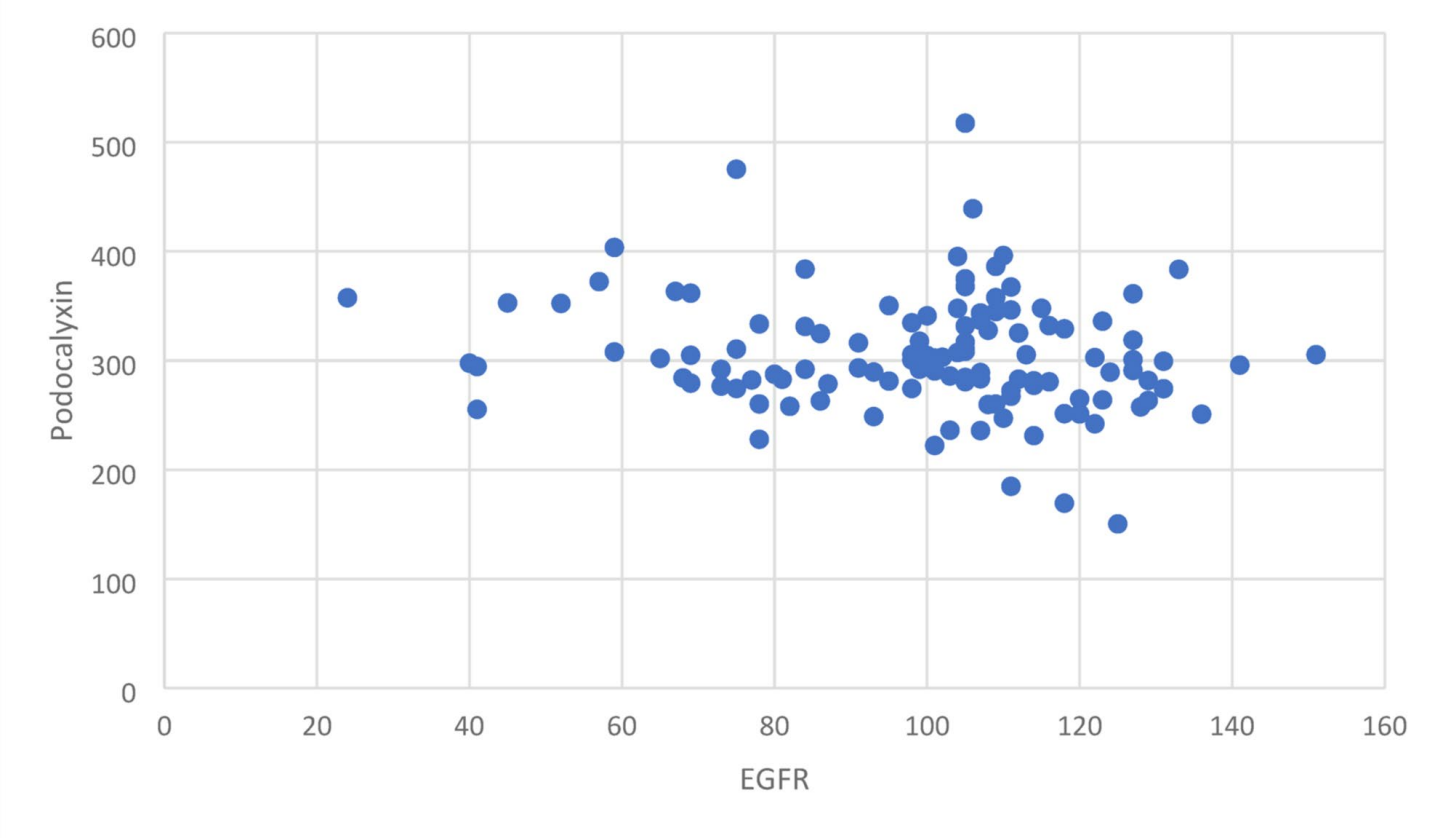
Scatter plot between urinary podocalyxin and eGFR

Another scatter plot was drawn to study the correlation between urinary podocalyxin and eGFR levels. It showed no relationship between the two parameters (*p* > 0.05). This is shown in Fig. 2.

**Fig. 3:**
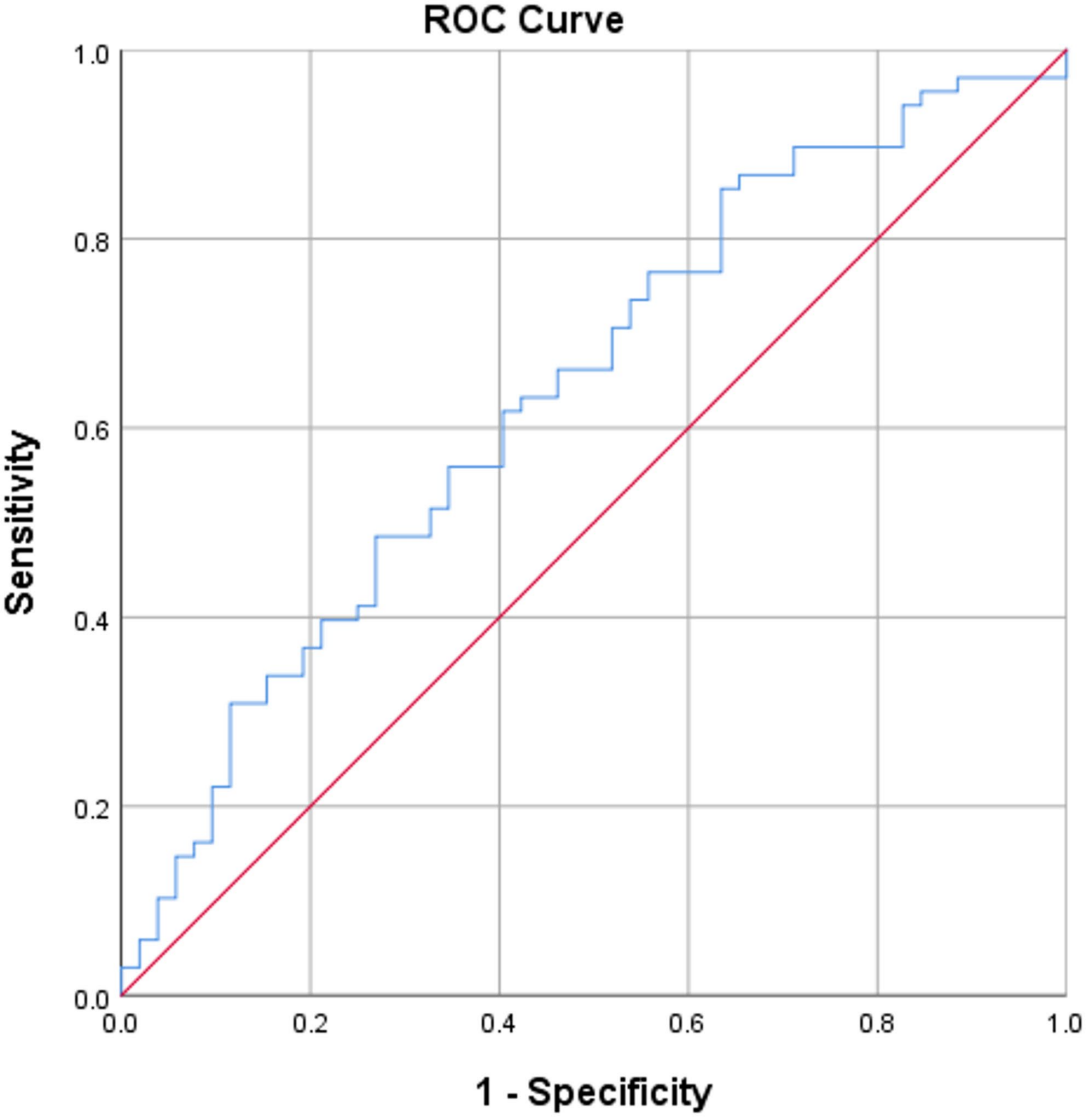
ROC curve for urinary podocalyxin and UACR

The receiver operator characteristic (ROC) curve was plotted to assess the utility of urinary podocalyxin as a marker for predicting diabetic nephropathy in relation to UACR. With a cut-off value of 275.56 ng/mL, urinary podocalyxin showed a sensitivity of 85% and specificity of 64% (area under curve = 0.637, 95% confidence interval 0.537–0.737, *p* = 0.003). This is shown in Fig. 3. The cut-off value was determined based on the median urinary podocalyxin value in the control subjects (275.8 ng/mL).

**Fig. 4:**
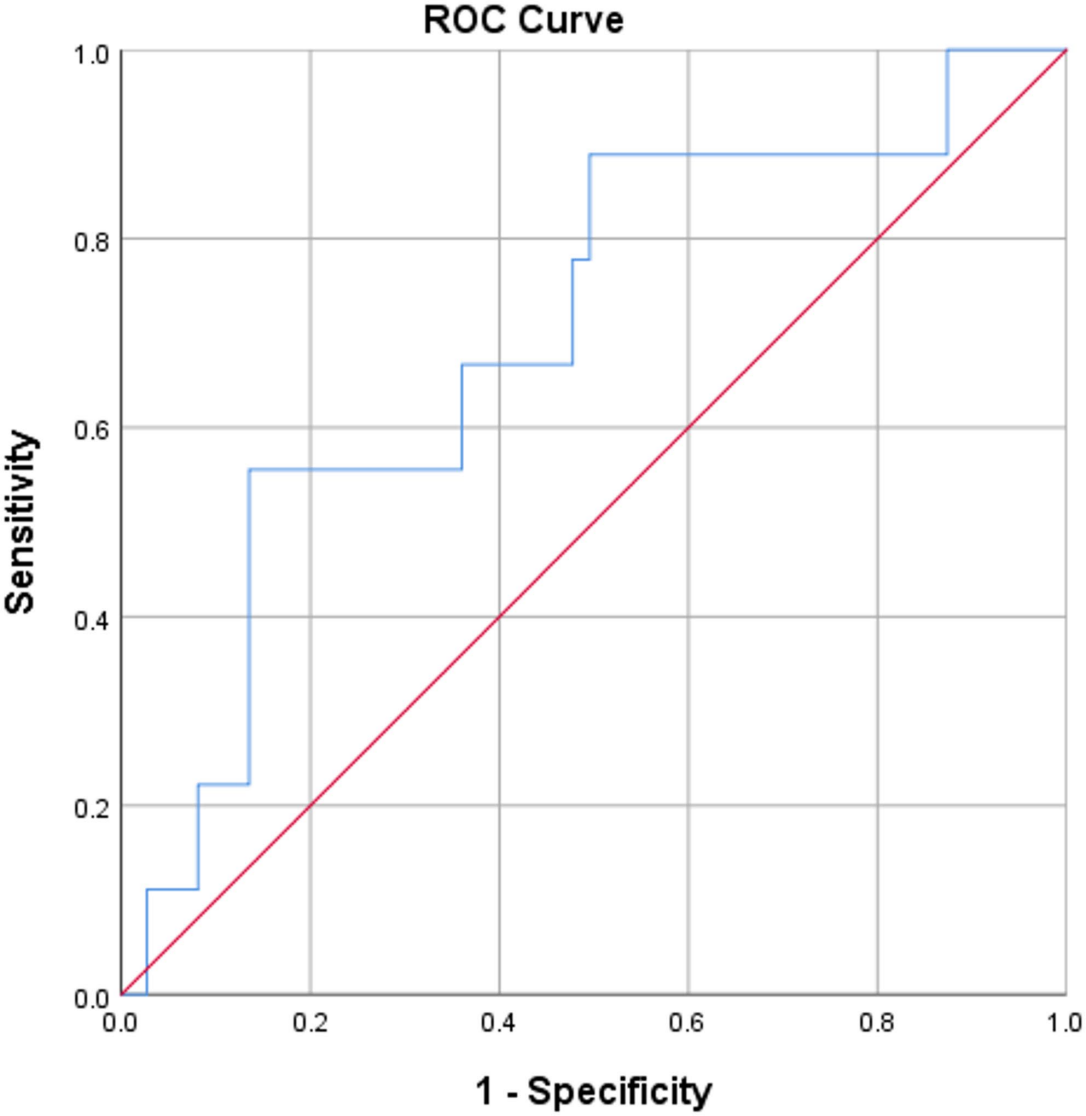
ROC curve for urinary podocalyxin and eGFR

Another ROC curve was plotted between urinary podocalyxin and eGFR. A cut-off value of 273.38 ng/mL for urinary podocalyxin had a sensitivity of 89% and specificity of 78% (area under the curve = 0.69, 95% confidence interval 0.52–0.88, *p* = 0.069). This is shown in Fig. 4.

## Discussion

Diabetic kidney disease (DKD) is clinically diagnosed in a patient with long-standing diabetes (duration more than ten years) having albuminuria and a lower estimated glomerular filtration rate (eGFR) in the absence of other causes of renal impairment [11]. The American Diabetes Association (ADA) 2020 standards of care recommend that patients with newly diagnosed type 2 diabetes mellitus should be screened for DKD with urinary albumin and eGFR [12]. However, recently, it has also been recognised that the reduction in eGFR can occur with normal urinary albumin excretion in both type 1 and type 2 diabetes mellitus [13].

Our study found that urinary podocalyxin values were elevated in diabetic subjects compared to the control group. A study by Nakamura et al. found urinary podocalyxin to be absent in nondiabetic subjects and diabetic patients with normoalbuminuria, while it was elevated in diabetic patients with micro and macroalbuminuria [14]. This value difference could have been due to how urinary podocalyxin levels were measured. In our study, urinary podocalyxin was measured using the ELISA technique, whereas in the study by Nakamura et al., it was measured using the immunofluorescence technique.

Our study also found that urinary podocalyxin levels were more elevated in diabetic subjects with microalbuminuria compared to those with macroalbuminuria. The exact reason for the higher urinary podocalyxin level in subjects with microalbuminuria is unclear. However, the possible explanation for this could have been the use of ELISA kits for the estimation of urinary podocalyxin. In our study, urinary podocalyxin values were elevated in diabetic subjects with normoalbuminuria compared to the control group. A study by Irena et al. showed similar results [15]. This suggests that urinary podocalyxin levels were elevated before the onset of microalbuminuria in diabetic nephropathy.

Podocalyxin is present in the glomerulus and is responsible for its structural integrity. Renal injury in diabetes mellitus leads to the appearance of podocalyxin in urine before that of albumin. Hence, urinary podocalyxin might be useful as an early marker for diabetic nephropathy. A study by Shoji et al., also reported that urinary podocalyxin levels could serve as an early marker for diabetic nephropathy and as a treatment target for the condition [16].

Our study found that for a cut-off value of 275.56 ng/mL, urinary podocalyxin had a sensitivity of 85% and specificity of 64% in predicting diabetic nephropathy. A study by Zheng et al. showed that the area under the curve was 0.753 (95% confidence interval, 0.623–0.883) for urinary podocalyxin mRNA levels, demonstrating the highest diagnostic value. The log-transformed threshold providing optimal sensitivity and specificity for podocalyxin mRNA was 23.24. Using the cut-off value of 23.24 derived from the data, podocalyxin mRNA levels predicted diabetic nephropathy with a sensitivity of 81.4% and a specificity of 62.5% [17]. In another study by Ghorab et al., the area under the curve for the validity of urinary podocalyxin was measured to be 0.996 [18].

In a study by Li JJ et al., other biomarkers for diabetic nephropathy, like cystatin-C and α2-macroglobulin, were studied along with urinary podocalyxin. Urinary α2-macroglobulin was elevated in patients with diabetic nephropathy, showing that urinary levels of α2-macroglobulin increased with aggravation of the condition. Their study evaluated the combination of urinary macroglobulin and podocalyxin for the diagnosis of diabetic nephropathy. The combination of the two biomarkers was found to be very efficacious, with the combined sensitivity and specificity at 86% and 76%, respectively, in diagnosing diabetic nephropathy [19]. Another study done by Ye et al., studied the correlation between urinary podocalyxin positive-element, urinary albumin, serum cystatin-C, and serum creatinine. Their study showed that urinary podocalyxin positive-element correlated with urinary albumin, serum cystatin-C, and serum creatinine. ROC curve analysis indicated that the area under the curve (AUC) of urinary podocalyxin positive-element was higher than that of serum cystatin-C and serum creatinine for discriminating nephropathy between diabetic patients and healthy controls [20].

Another study by Hara M et al., evaluated urinary podocalyxin as a marker for podocyte injury in patients with type 2 diabetes mellitus. Urinary podocalyxin was measured by immunofluorescence, immunoelectron microscopy and western blotting techniques. Urinary podocalyxin was higher than the cut-off value in 53.8% of patients with normoalbuminuria, in 64.7% of patients with microalbuminuria and in 66.7% of patients with macroalbuminuria [21].

Our study also showed a significant positive correlation between urinary podocalyxin and urinary albumin-creatinine ratio (UACR). A study by Ashraf et al. also found a significant positive correlation between urinary podocalyxin and albuminuria (*r* = 0.782, *p* < 0.001) [22]. This study indicated that urinary podocalyxin level increases as UACR increases.

Our study did not find a relationship between urinary podocalyxin and eGFR values. However, a study by Xie Y et al. showed that eGFR influenced the predictive ability of urinary podocalyxin in the diagnosis of early diabetic kidney disease [23]. The reason for the different findings between our study and their study is not clear.

## Limitations of the study

The sample size for the study was small. A single-spot urine sample was used to estimate urine albumin. Instead, a 24-hour urine albumin estimation would have given more accurate values. Age and gender distribution were not similar between the control and diabetic study groups. Being a cross-sectional study, the temporal association between urinary podocalyxin and diabetic nephropathy could not be determined. Glycemic status and drug history of study subjects were not elicited.

## Conclusion

Urinary podocalyxin levels were elevated in type 2 diabetic patients compared to non-diabetic subjects. There was a positive correlation between urinary podocalyxin values and urine albumin-creatinine ratio (ACR) in diabetic subjects. However, there was no correlation between urinary podocalyxin and the estimated glomerular filtration rate (eGFR). Hence, further clinical research is needed before the use of urinary podocalyxin as a biomarker is possible.

## Data Availability

No datasets were generated or analysed during the current study.
